# Artificial Intelligence in the Intensive Care Unit: Current Evidence on an Inevitable Future Tool

**DOI:** 10.7759/cureus.59797

**Published:** 2024-05-07

**Authors:** Vinay Suresh, Kaushal K Singh, Esha Vaish, Mohan Gurjar, Anbuvanan Ambuli Nambi, Yashita Khulbe, Syed Muzaffar

**Affiliations:** 1 General Medicine and Surgery, King George's Medical University, Lucknow, IND; 2 General Medicine, King George's Medical University, Lucknow, IND; 3 Internal Medicine, Mount Sinai Morningside West, New York, USA; 4 Critical Care Medicine, Sanjay Gandhi Postgraduate Institute of Medical Sciences, Lucknow, IND; 5 General Medicine and Surgery, Madras Medical College, Chennai, IND; 6 Critical Care Medicine, King George's Medical University, Lucknow, IND

**Keywords:** sepsis, nutritional support, mechanical ventilation, intensive care unit, delirium, critical care medicine, treatment outcome, ards, artificial intelligence

## Abstract

Artificial intelligence (AI) is a technique that attempts to replicate human intelligence, analytical behavior, and decision-making ability. This includes machine learning, which involves the use of algorithms and statistical techniques to enhance the computer’s ability to make decisions more accurately. Due to AI’s ability to analyze, comprehend, and interpret considerable volumes of data, it has been increasingly used in the field of healthcare. In critical care medicine, where most of the patient load requires timely interventions due to the perilous nature of the condition, AI’s ability to monitor, analyze, and predict unfavorable outcomes is an invaluable asset. It can significantly improve timely interventions and prevent unfavorable outcomes, which, otherwise, is not always achievable owing to the constrained human ability to multitask with optimum efficiency. AI has been implicated in intensive care units over the past many years. In addition to its advantageous applications, this article discusses its disadvantages, prospects, and the changes needed to train future critical care professionals. A comprehensive search of electronic databases was performed using relevant keywords. Data from articles pertinent to the topic was assimilated into this review article.

## Introduction and background

Artificial intelligence (AI) is a technique that replicates human intelligence, analytical behavior, and decision-making ability in machines. AI should not be confused with machine learning (ML) or deep learning (DL), which work on the principle of identifying recurring patterns in a dataset.

AI and ML have aided clinicians in various labor-intensive tasks such as rapid diagnosis and prediction of patient outcomes, risk stratification, optimizing resource allocation, and continuous patient monitoring. Recently, the role of AI in emergency and intensive care settings has become a topic of interest. AI can automate critical patients’ monitoring and predict prognosis [1,2]. However, barely any literature exists to discuss such possibilities, especially in lower-middle-income countries like India. This review article discusses numerous applications of AI in critical care medicine (CCM), its disadvantages, the future, and possible changes in the training of future critical care specialists.

## Review

Applications of AI in CCM

The most revolutionary application of AI in CCM would involve early disease identification, predicting disease evolution, individualized treatment, and optimizing the allocation of resources [3].

AI in the intensive care unit (ICU)

AI and Respiratory System

AI has optimized mechanical ventilation tailored to the patient’s needs. A study aimed to prevent patient demand for ventilator supply asynchrony built a predictive model via supervised learning. The algorithms trained the models using flow rate and airway pressure to estimate resistance (R) and compliance (C) values based on labeled data read from ventilation sensors. Using a regression model, the algorithm reached 99.4% accuracy in detecting R-C levels [4]. Similarly, Liu et al. developed a two-stage AI system that could predict the optimal time to wean patients from ventilatory support in the ICU [5].

Acute respiratory distress syndrome (ARDS) phenotyping: A study by Calfee et al. points out how the heterogeneous nature of ARDS translates to a possible lack of understanding of the biological mechanisms. Using latent class analysis, the study identified two phenotypes of ARDS: P1 and P2. Higher plasma levels of IL-6, IL-8, soluble tumor necrosis factor receptor 1, and plasminogen activator inhibitor 1, as well as clinical findings such as higher heart rate, higher total minute ventilation, and lower systolic blood pressure in P2 as compared to P1, reflected significant differences. P1 was more likely to have trauma-associated ARDS and less likely to have sepsis-associated ARDS [6]. Another ML model developed recently by Sinha et al. was found to have equivalent performance [7]. Similar models may be quintessential in the subphenotyping of other diseases with underlying clinical heterogeneity, e.g., sepsis, and impact their clinical management.

Table 1 summarizes the recent studies on AI with respect to the respiratory system.

**Table 1:** Role of AI and ML in ICU patients: recent studies related to respiratory system AI, artificial intelligence; ARDS, acute respiratory distress syndrome; DBP, diastolic blood pressure; GCS, Glasgow Coma Scale; HR, heart rate; ICU, intensive care unit; IOT, Internet of Things; ML, machine learning; mPaw, mean airway pressure; MV, minute ventilation; Ppeak, peak inspiratory pressure; PSL, pressure support level; PSL volume, tidal volume with pressure support; RR, respiratory rate; SBP, systolic blood pressure; SBT, spontaneous breathing trial; SpO2, oxygen saturation; TISS, Therapeutic Intervention Scoring System

SN	Purpose	Salient points	Author, year, and country	Reference
1	To get real-time data on respiratory resistance (R) and compliance (C) continuously and in real time	Input variables for the model: flow rate and airway pressure accuracy of 63.9-93.1% (99.4% using the regression model)	Hezarjaribi et al. (2018), USA	[4]
2	To build two-stage predictive models, namely, the try-weaning stage and the weaning MV stage, to determine the optimal timing of weaning from mechanical ventilation for ICU intubated patients	Stage 1: 25 features: primary patient data of age, APACHE II, TISS score, and the first and last IoT data of the respirator consisting of FiO2, PEEP, respiratory rate, MV, Ppeak, mPaw, SpO2, Vte, HR, SBP, and DBP; accuracy for Stage 1: 68.6-77.4%. Stage 2: 20 features: primary data consisting of age, APACHE II score, and TISS score and the last respirator IoT data before extubation consisting of FiO2, PEEP, RR, MV, Ppeak, mPaw, SpO2, PSL, PSL volume, body temperature, HR, SBP, DBP, GCS eye-opening, GCS motor response, SBT count during support mode, and sputum suction count within 24 hours before extubation; accuracy for Stage 2: 64.9-84.2%	Liu et al. (2022), Taiwan	[5]
3	To identify the ARDS phenotype (hyperinflammatory or hypoinflammatory)	Model 1 (three variables): IL-8, bicarbonate, and protein C; sensitivity for Model 1: 74-84%. Model 2 (four variables): IL-8, bicarbonate, protein C, and vasopressor use; sensitivity for Model 2: 82-91%	Sinha et al. (2020), USA and UK	[7]

AI and Sepsis

An algorithm called Artificial Intelligence Sepsis Expert analyzed factors such as pulse rate and mean arterial pressure, along with structured electronic medical record (EMR) data, to predict sepsis four hours in advance with an area under curve (AUC) of 0.85 [8]. Another cohort study used an AI tool termed Long Short-Term Memory (LSTM). It fed the information of nearly 59,000 patients from the MIMIC III database, including vital signs, lab tests, drug intake, physician notes, and clinical outcomes. Its AUROC and hours before onset were better than or on par with current models. It managed to pick up patients close to 20 hours quicker than a Cox proportional hazards model and provided the same target definitions and features [9].

One cohort study conducted over 92 ICU patients assessed the levels of ILs like IL-beta, IL-6, IL-8, IL-10, and other cytokines such as TNF-α, Fas ligand, and CC chemokine ligand 2 mRNA, fed it to a neural network, and predicted patient outcomes with an accuracy of 94.55% and high sensitivity [10].

Another study by Yuan et al. used the Sepsis-3 definition and evaluation using the Sepsis-related Organ Failure Assessment (SOFA) score to include 106 features, such as organ dysfunction and status of infection, to provide a timely diagnosis of sepsis, with an accuracy exceeding 80% [11].

An AI tool with natural language processing capability, Sepsis Early Risk Assessment, utilized clinician’s journals (unstructured data) and EMRs (structured data) for accurate sepsis risk assessment and was capable of warning clinicians two days in advance. SERA outperformed the hospital’s physicians and human-based scoring methods such as quick SOFA (qSOFA), Modified Early Warning Score, systemic inflammatory response syndrome, and SOFA [12].

Table 2 summarizes the recent studies on AI with respect to sepsis.

**Table 2:** Role of AI and ML in critically ill patients: recent studies related to early prediction of sepsis and septic shock ABG, arterial blood gas; AI, artificial intelligence; AISE, Artificial Intelligence Sepsis Expert; AUC ROC, area under ROC curve; BP, blood pressure; DBP, diastolic blood pressure; FASL, Fas ligand; FiO2, fraction of inspired oxygen; GCS, Glasgow Coma Scale; Hb, hemoglobin; HR, heart rate; LSTM, Long Short-Term Memory; ML, machine learning; pCO2, partial pressure of arterial CO2; pO2, partial pressure of arterial oxygen; SBP, systolic blood pressure; SERA, Sepsis Early Risk Assessment; SpO2, oxygen saturation

SN	Purpose	Salient points	Author, year, and country	Reference
1	Early prediction of sepsis (AISE algorithm)	Input variables: 10 clinical features evaluating the cardiovascular and respiratory systems, 25 general laboratory features, five ABG-related features, 19 demography and history-related features, and six high-resolution dynamic features; accuracy: 63-67%	Nemati et al. (2018), USA	[8]
2	Early prediction of sepsis	Input variables: leukocyte expression of cytokines: IL-1β, IL-6, IL-8, IL-10, MCP-1, TNF-α, and FasL; accuracy: 94.55%	Lukaszewski et al. (2008), UK	[10]
3	Early prediction of sepsis	Input variables: age, gender, BP, HR, temperature, SpO2, respiratory rate, total white cell, culture results, lactate, high-sensitivity CRP, procalcitonin, ABG, vasopressor use, antibiotic use, clinical notes describing clinical status, communication, laboratory tests, non-clinical status, social relationships, symptom, and treatment; accuracy: 82% ± 1%	Yuan et al. (2020), Taiwan	[11]
4	Early detection of septic shock (LiSep LSTM)	Input variables: age, arterial pH, DBP, FiO2, GCS, HR, mean arterial BP, platelet count, respiratory rate, Riker Sedation-Agitation Scale, SBP, SpO2, bicarbonate level, BUN, creatinine, hematocrit, Hb, potassium, pCO2, pO2, WBC count, fluid input, antibiotics, and urine output; AUC ROC: 0.8306 (95% CI: 0.8236, 0.8376)	Fagerström et al. (2019), Sweden	[9]
5	Early detection of septic shock (SERA algorithm)	Input variables: vitals, investigations, treatment, and other clinical notes of admission, progress, ICU consult, pharmacy, and allied health; AUC ROC: 0.94	Goh et al. (2021), Singapore	[12]

AI and COVID-19

The following AI models have demonstrated promising results that can potentially alleviate the burden on healthcare infrastructure by facilitating early detection, rapid risk assessment, and triage of COVID-19 cases.

An AI-driven triage system named CURIAL was used in a hospital in the UK for COVID-19 screening. The technique employed routine tests available within an hour of admission. With algorithms optimized for generalizability and speed, the AUC was 0.858-0.881 [13]. Another model based on Least Absolute Shrinkage and Selection Operation regression and AUROC scores of 0.841 and 0.938 with a 100% recall score considered age, IL-6, systolic blood pressure, monocyte ratio, and fever classification for a quicker diagnosis in cases where COVID-19 was suspected [14].

Yet another deep convolutional neural networks-based model used an image classifier to read chest X-rays and a gradient boosting model to learn from other routinely collected clinical variables to deduce a deterioration risk with an AUC of 0.786, thereby aiding in critical decision-making [15]. Another model, named CoviDet, was shown to diagnose COVID-19 with only a chest CT of the patient, with an AUC of 0.98, surpassing the assessment of radiologists. The same model made use of serial CTs in autosegmentation analyses to monitor the clinical course of the patient [16].

Another study used federated learning to train AI models and came up with the EMR Chest X-ray AI Model (EXAM). In this model, the oxygen needs of symptomatic COVID-19 patients are predicted by using heterogeneous variables, including laboratory results and vital signs, besides chest X-rays. EXAM showed an AUC >0.92 at 24 and 72 hours from the time the patient first presented to the emergency room [17].

One study used the traditional Cox model, which uses regression analysis to identify a relationship between a covariate, such as clinical traits, and the chance that an event will occur (such as comorbidity or mortality). The Cox model was integrated with more extensive techniques in order to accurately predict the clinical outcome in cases of COVID-19. With a C-index and AUC of 0.894 and 0.91, respectively, these values were higher than those of the classical Cox model [18].

A prospective observational study by Maheshwarappa et al. revealed that the AI-enhanced portable ultrasound gadget, when compared to its conventional counterpart, yielded significantly superior results when assessing the cardiopulmonary system of COVID-19 patients, in both the contact risk of the healthcare workers as well as the time taken to make a diagnosis. In comparison to the conventional method, which took 20 (17-22) minutes for each examination, employing portable ultrasound took nine (8.0-11.0) minutes on average (P = 0.001) [19].

AI and Renal System

Renal diseases are adding to the ever-increasing health issues globally, with diabetes being the leading cause. The development of end-stage renal disease (ESRD) can be effectively halted if clinicians intervene at the appropriate time. Currently, AI is targeted at alerting, assisting with diagnosis, guiding therapeutic measures, and predicting patient outcomes. In 2014, NHS England recommended using a standardized acute kidney injury (AKI) algorithm to detect AKI across hospitals in the UK [20]. Tomašev et al.’s AI model predicted 55.8% of AKI incidences and 90.2% of all such patients in whom dialysis became necessary, out of 703,782 individuals [21]. Another retrospective review of 8,800 patients who underwent contrast administration was done by Yin et al. to develop an AI-Random Forests (AI-RF) model to predict contrast-induced nephropathy [22].

With the available alerting AI tools, clinicians can intervene before the patient develops ESRD. A pilot study from Australia revealed that an AI software program could pick out at-risk patients and help choose appropriate screening tests for ESRD [23].

A computer-aided diagnosis algorithm is a computer image processing technology that utilizes clinical images to accurately identify a lesion. The automated Deep Feature Classification tool helps differentiate malignant renal cell carcinoma from benign renal neoplasms such as angiomyolipoma and oncocytoma based on abdominal radiographs [24].

In 2015, Barbieri et al. used a multilayer perceptron to build a model for predicting the individualized therapeutic dose for erythropoietin-stimulating agent therapy for managing anemia in CKD patients. Decreased hemoglobin fluctuations in these patients reduced the cost of treatment and the frequency of transfusions, hospitalizations, and cardiovascular events [25].

Wearable dialysis devices were developed by Hueso et al. by combining AI and regenerative medicine technology to conduct continuous dialysis and remove toxins efficiently with minimal effect on hemodynamics [26]. Although still under development, the implantable renal assist device, also known as the bionic or artificial kidney, is yet another remarkable innovation that mimics the kidney’s morphology and function [27].

AI can also predict the prognosis on a case-by-case basis; the RF model by Dagliati et al. has the highest performance. It showed that variables like age, gender, duration, obesity, hypertension, glycemic control, and tobacco consumption had a greater influence on whether complications appeared in type 2 diabetes mellitus patients than other variables [28].

Table 3 summarizes the recent studies on AI with respect to AKI.

**Table 3:** Role of AI and ML: recent studies related to AKI AI, artificial intelligence; AKI, acute kidney injury; eGFR, estimated glomerular filtration rate; HDL, high-density lipoprotein; INR, international normalized ratio; LDL, low-density lipoprotein; ML, machine learning; P-LCR, platelet larger cell ratio; RDW, red cell distribution width

SN	Purpose	Salient points	Author, year, and country	Reference
1	Early prediction of AKI within a clinically actionable window of up to 48 hours in advance	Input variables: outpatient visits, admissions, diagnoses, procedures, laboratory results (biochemistry, hematology, cytology, toxicology, microbiology, histopathology, etc.), medications and prescriptions, orders, vital signs, health factors, and note titles; AUC ROC: 0.921 (any stage AKI), 0.957 (stage 2/3 AKI), and 0.980 (stage 3 AKI); sensitivity at 20% precision level: 76.7% (any stage AKI), 82.0% (stage 2/3 AKI), and 91.2% (stage 3 AKI)	Tomašev et al. (2019), USA	[21]
2	Early prediction for contrast-induced nephropathy before patients’ exposure to contrast media	Input variables: baseline eGFR, RDW, triglycerides, most recent serum creatinine before the procedure, HDL, total cholesterol, LDL, blood urea, P-LCR, serum sodium, platelet crit, INR, and blood glucose; accuracy: 80.80%	Yin et al. (2017), China	[22]

AI and Cardiovascular System

AI has been extensively used for timely diagnosis, clinical and interventional care, prognostication, and pharmacological research in cardiovascular medicine. The AI-Clinical Decision Support System (AI-CDSS) employs a hybrid approach to diagnosing coronary heart disease (CHD) that is most useful, especially when the specialists are unavailable or unsure of the diagnosis [29].

Early diagnosis of CHD using rough sets and logistic regression methods plays a vital role in choosing the management protocol specific to heart failure (HF) patients and reducing the high mortality and morbidity associated with it. AI can also be used as an assisting resource by clinicians in their daily practice to reduce medication errors [30].

A study applied a neural network algorithm for diagnosing HF in 40 individuals with 85% accuracy using clinical data such as age, gender, blood pressure, and smoking history [31]. HeartModel is a software that analyzes echocardiographic parameters such as chamber volumes and ejection fractions (EFs) to determine disease status and treatment options [32]. The V-LAP is the first battery-less cardiac monitoring device that helps physicians monitor patients’ LAP continuously, diagnose HF before symptom onset, and provide remote HF care [33].

The AI-augmented electrocardiogram (AI-ECG) has also been proven to be more accurate in detection and prognostics. A study showed that the AI-ECG can be used in the detection of left ventricular systolic dysfunction (LVSD) in patients admitted to the critical ICU, both with and without atrial fibrillation [34]. Another study showed that AI-ECG, when used in mortality risk stratification for LVSD, showed a higher probability of mortality association than what could be explained by a reduced left ventricular EFs alone. Hence, it was more helpful in prognosticating cases with insignificant LVSD, thus showing the importance of subclinical myocardial disease [35].

Heart disease development is greatly influenced by genetic predisposition. Comprehensive genome-wide association research can be used to predict advanced coronary artery calcification using DL networks. Furthermore, AI has been found to be 80% accurate, which is more than clinicians (60%), in predicting the five-year survival rate for individuals with cardiovascular disease [36].

Feasible Artificial Intelligence with Simple Trajectories for Predicting Adverse Catastrophic Events (FAST-PACE) is a model used to predict the occurrence of respiratory failure or cardiac arrest up to six hours before its onset; the AUROC curve of this model is 0.869 and 0.886 for the two outcomes, respectively, six hours before their onset [37].

AI predictive models can predict the risk of future hospitalizations. For instance, one study used RFs to predict unplanned all-cause 30-day rehospitalization for congestive HF patients based on the diagnosis, procedure, a combination of both, and demographic data with an AUC of ≥0.8 [38]. Multiple studies have utilized the Outcome and Assessment Information Set (OASIS) to identify risk factors for rehospitalization. Using the OASIS-C version yielded a concordance (c) statistic (an AUC equivalent) of 0.59 [39].

AI in Trauma

AI has been put to effective use in trauma care as well. Globally, skeletal injury is one of the primary complaints of individuals brought to a critical care physician. Radiography is the imaging modality of choice for these patients worldwide. Interpretation of radiographs remains a challenging affair requiring significant skill and experience. In the setting of a lack of radiologists, critical care physicians must act swiftly before a radiologist’s opinion is available, risking a mistake in reading the radiograph. Missed fractures contribute to over three-fourths of diagnostic errors in the emergency department [40]. One study demonstrated how an AI tool assisted critical care physicians in detecting fractures of the appendicular skeleton in adult patients, with a stand-alone AUC of 0.94 [41]. A similar study on appendicular skeletal fractures in pediatric patients revealed a 10% increase in sensitivity with no significant reduction in specificity when radiologists made use of an AI tool [42]. However, these retrospective studies lacked the cognitive bias associated with a trauma ward setting and also ignored the clinical data (physical examination and medical history of the patient), analyzing only the ability of the AI and radiologists to read the radiographs.

AI and Treatment Outcomes

AI has also been used to predict patient morbidity and mortality in hospital-admitted patients. The Early Mortality Prediction for Intensive Care Unit Patients used RF, predictive decision trees, probabilistic Naive Bayes, and rule-based Projective Adaptive Resonance Theory models to predict patient mortality. It outperformed standard scoring systems (SOFA, SAPS-I, and APACHE-II), the National Early Warning Score (NEWS), and qSOFA in terms of AUROC and time [43].

AutoTriage, a diagnostic algorithm devised by Calvert et al., uses eight clinical variables from electronic health records to make a prediction of patient mortality. The AUROC value for the algorithm was found to be 0.88 [44]. Another study used both clinical parameters, medications, and laboratory tests to predict the duration of hospital stay and chances of mortality in cases of COVID-19 and showed good reliability with a coefficient of determination R2 of 49.8%. The clinical parameters in the model included initial diagnosis and intubation or ventilatory support; medications included analgesics and anti-inflammatory, antimicrobials like azithromycin and antivirals, and vitamin C; and lab tests included urea, platelet count, D dimer, potassium, and hemoglobin [45].

Table 4 summarizes the recent studies on AI with respect to outcomes.

**Table 4:** Role of AI and ML in critically ill patients: recent studies related to life-threatening events, including cardiac arrest or mortality AI, artificial intelligence; AUC ROC, area under ROC curve; DBP, diastolic blood pressure; FAST-PACE, Feasible Artificial Intelligence with Simple Trajectories for Predicting Adverse Catastrophic Events; ICU, intensive care unit; ML, machine learning; SBP, systolic blood pressure; SpO2, oxygen saturation

SN	Purpose	Salient points	Author, year, and country	Reference
1	To predict adverse events (cardiac arrest or acute respiratory failure) one hour to six hours prior to their occurrence without lab data (FAST-PACE)	Input variables: pulse rate, SBP, DBP, respiratory rate, SpO2, body temperature, treatment history, recent surgical history (within one week), and current health status (ASA classification); accuracy: cardiac arrest: 77.9%, 74.5%, 80.1%, and 75.1%; respiratory failure: 78.2%, 75.3%, 79.8%, and 75.4%	Kim et al. (2019), Korea	[37]
2	Early mortality prediction for ICU patients	Input variables: demographics, physiological variables, vital signs, and laboratory test variables; AUC ROC: 0.82	Awad et al. (2017), UK	[43]

AI and Nutritional Support

AI, through variable data integration, can make individualized decisions regarding a critically ill patient’s optimal nutrition status. The computer-assisted decision support systems (CDSS) have been shown to increase caloric and protein goal compliance by more than 50% compared to the cohort with unmonitored nutritional care. However, hospital mortality, ICU-acquired infections, and lengths of stay did not statistically improve with the use of CDSS [46].

Malnutrition in older hospitalized patients is not well recognized and has dreadful effects on their prognosis, with prolonged hospitalization and rehabilitation periods. Another AI tool has been used to estimate the calorie and macronutrient intake of geriatric patients based on images of their meals before and after consumption in the setting of a standardized hospital kitchen menu database. The automated system was found to have higher accuracy than the estimation by nursing staff, who were found to often overestimate energy and protein intake. Therefore, there is an opportunity for AI to better pick up malnourished patients in the hospital with accurate monitoring of their energy and macronutrient intake, especially when they do not complete their full meals [47].

Role of AI in Delirium

Delirium is often undocumented and underdiagnosed, despite being associated with a multitude of identifiable risk factors. One study utilized an RF ML approach to establish a correlation between patient characteristics and 4 ‘A’s Test (4AT) scores, a convenient screening tool for delirium [48]. The variables that correlated significantly with the 4AT score were age, physical restraint, dementia, diabetes, ward type, educational level, and gender. However, this study lacks generalizability as it was conducted in specific ward settings, where risk factors for delirium are already high.

Another retrospective study on the efficacy of an AI-based algorithm, the Natural Language Processing Confusion Assessment Method (NLP-CAM), in detecting delirium based on EMRs of COVID-19 patients admitted to hospitals revealed an increased rate of detection by the NLP-CAM algorithm of 80% as compared to physician diagnosis of 55% [49].

Other benefits of AI in the ICU

Apart from aiding in diagnosis, optimal resource allocation and crowd management can become game changers in the ED, directly impacting the efficacy, quality, and cost of care. Sun et al. proposed using forecasting AI models for staff roster planning and resource allocation [50].

Triaging in the ED can be another effective application of AI, enhancing patient movement and resource allocation. An algorithm developed in a university hospital was introduced to the 2019 American College of Surgeons Clinical Congress. It assimilated 87 clinical factors and 15 unique criteria to gauge whether ICU admission within two days of a surgical procedure was appropriate or not. A comparison was also made with the results of a questionnaire given to the clinicians. The AI was more accurate in triaging patients - 41 of the 50 patients (82%) - in comparison to surgeons at 70% (35 patients), critical care physicians at 64% (32 patients), and anesthetists at 58% (29 patients) [51].

AI can be used to generate alarms for the critical conditions of patients. An alarm system designed by Sukuvaara et al. helps in detecting decreased or increased circulatory volume, elevated blood pressure, LVSD, and impaired ventilation based on the principle of rule-based expert systems [52]. When integrated with ML, the actual patient data can update the pre-existing database [53]. Laursen developed software based on Bayesian networks that, through continuous comparison of various clinical parameters and their changes, may be able to anticipate cardiac events [54]. AI tools are also utilized in ventilators to trigger alarms for deteriorating patients [55].

Educating and training healthcare professionals about the latest practices is crucial for the evolution of medicine. AI could possibly act as a tool to teach clinical skills with limited resources to meet the increasing demand for training professionals. One study predicted a 47.5% increase in the use of AI in the education industry in a single year [56]. With this comes the concern that AI may replace the teaching of clinical skills by trained professionals. However, clinicians must still spend quality time at bedside training programs, as there is no substitute for the human touch in clinician-patient interactions.

The AI also has a virtual nurse model that can assist patients at home [57], thus bridging the shortage of nursing care professionals.

Drawbacks of AI applications in CCM

Although various studies have supported the application of AI models in the critical care setting, the most glaring limitation is their insufficient sample size and insufficient validation of predictions. Results derived from such studies are riddled with two major problems: over-fitting, a potential problem with any prediction model, and model optimism [58]. An AI model built on the limited data set of one institution will fail when applied to a different institution due to technical differences and variations in local practices and patient characteristics. Ideally, ICU data across all institutions must be collected to create a large and diverse data set.

On the same note, if large and diverse patient data goes into an AI model, accessing such sensitive information means challenging patient privacy [59]. This threat to patient privacy can cripple AI applications and require the implementation of strong safety regulations.

Several studies have proposed AI’s use as a decisive tool in the clinical management of COVID-19 patients. However, the information generated by these studies is grossly insufficient. For instance, several studies propose using AI for the detection and determination of the severity of COVID-19 patients based on radiographic imaging. However, this has many drawbacks, such as the limited capacity to rule out different differential diagnoses based on radiographic images alone; also, several symptomatic patients have normal radiographs. Suri et al. have discussed the vast potential for improvement in AI-assisted diagnostics in the COVID-19 scenario [60].

Another point of concern is that, as of today, clinics use both paper and electronic documentation. This can lead to incomplete and inconsistent datasets for an AI algorithm, potentially causing inappropriate interventions and threatening patient safety.

Another issue with AI is model transparency. A physician can explain their thought processes. This helps in identifying potential areas for mistakes. However, an AI is notoriously famous for its “black box” nature (lack of transparency) [1]. There have been instances where a lack of transparency has inadvertently led to discrimination or inappropriate outcomes. For example, a widely used American hospital algorithm was noted to have an unintentional racial bias when allocating healthcare resources to black patients [61]. There are other ways an AI algorithm can exacerbate social and cultural inequalities. Most of the data used in AI training does not include enough relevant social factors, such as ethnicity or socioeconomic status, to account for all racial minorities properly.

AI applications will also meet several ethical dilemmas, such as end-of-life care. Can an AI adequately process the inputs from the patient, the surrogate, the caretakers, the family, and the physicians to generate the most relevant output in a critical care setting?

Lastly, most studies have focused on a narrow range of AI applications, resulting in fewer AI applications eventually being considered for clinical use. The application of AI in clinical settings today is largely limited to diagnostic and prognostic predictive models. Even though studies have proven AI to be superior to clinicians in this respect, the evidence is not strong, as most of these studies are retrospective analyses carrying a high risk of bias. Controlled clinical trials with adequate human comparators are required. Additionally, one must not forget the practical aspects of technology implementation: convenience, training, costs, patient hesitancy, etc.

Future prospects

It is evident from the wide implications of AI in ICUs that AI is revolutionizing the way we deliver critical healthcare. This may raise an important question, i.e., will AI algorithms replace healthcare providers in the ICUs, making critical care doctors obsolete soon?

Before answering this query, we must consider that, although these digital algorithms reduce manual efforts and errors, they are flawed and have disadvantages, as discussed above. Also, it is a great misconception that a critical care provider only stabilizes patients. The real job is much more than this. An experienced intensivist looks for numerous causes that could have led the patient to this condition, which digital algorithms cannot do. Thus, it is not possible to completely replace the manual workforce in the ICUs. However, we agree that many of the tasks in ICUs can be performed by digital algorithms in a timely and efficient manner. Moreover, AI will likely change the way critical healthcare providers perform their duties, so we should be better prepared for the healthcare demands of critical ICU patients.

Firstly, we need to revamp the whole training curriculum for critical care doctors. The new curriculum should incorporate the plan for extensive training of candidates to monitor and run these digital algorithms. The basics of the use of AI in healthcare should be taught to the candidates during their undergraduate education. Secondly, the country’s leading CCM and emergency medicine academic societies should step forward to ensure that AI is transforming critical healthcare in a better way and that AI is being utilized to its full potential by doctors-in-training to improve critical care. Lastly, the challenges faced by critical care doctors-in-training should be addressed properly by the advocates of AI and ML.

Thus, we are looking forward to a future of healthcare wherein digital algorithms will reshape the jobs of critical care doctors and may relieve them from repetitive, monotonous, and labor-intensive tasks in the ICUs to a significant extent. Moreover, there is a high probability that the augmentation of AI algorithms in critical care will enable us to save more lives and provide a forever-growing social and economic environment in which humankind will enjoy a higher standard of living.

## Conclusions

From a bird’s-eye view, the universal implementation of multimodal AI systems in critical care settings looks like a utopian vision at the moment. Attraction toward AI lies in the promise of amplifying physician performance in terms of efficiency and time and improving the patient experience (which is increasingly becoming an indispensable component of the healthcare system nowadays). Numerous studies can vouch for the fact that many AI models have even outperformed clinicians and conventional tools. However, the current AI applications are largely limited by the range of applications, clinical applicability, reproducibility, and lack of testing in complete real-world scenarios (uncountable and unpredictable). Superior quality evidence is necessary to assess the short- and long-term consequences thoroughly. AI’s supremacy lies in its propensity to update in real-time and organize the constantly incoming and changing data to generate an accurate outcome. If successfully adopted, AI can hold the torch for the future of CCM. However, one thing that we surely do not see AI replacing in the future is the classic human physician-patient relationship - the comfort of face-to-face conversation with a doctor.
